# De-hospitalization of the pediatric day surgery by means of a freestanding surgery center: pilot study in the lazio region

**DOI:** 10.1186/1824-7288-38-5

**Published:** 2012-02-01

**Authors:** Giovanni Mangia, Franco Bianco, Alma Ciaschi, Elisabetta Di Caro, Eufrasia Frattarelli, Giacinto Antonio Marrocco

**Affiliations:** 1 Departement of Anesthesia, San Camillo Hospital, Rome, Italy; 2 Departement Production Flow, Local Health Rome A, Italy; 3 Department Pediatric Surgery, San Camillo Hospital, Rome, Italy

## Abstract

**Background:**

Day surgery should take place in appropriate organizational settings. In the presence of high volumes, the organizational models of the Lazio Region are represented by either Day Surgery Units within continuous-cycle hospitals or day-cycle Day Surgery Centers. This pilot study presents the regional volumes provided in 2010 and the additional volumes that could be provided based on the best performance criterion with a view to suggesting the setting up of a regional Freestanding Center of Pediatric Day Surgery.

**Methods:**

This is an observational retrospective study. The activity volumes have been assessed by means of a DRG (Diagnosis Related Group)-specific indicator that measures the ratio of outpatients to the total number of treated patients (freestanding indicator, FI). The included DRGs had an FI exceeding the 3^rd ^quartile present in at least a health-care facility and a volume exceeding 0.5% of the total patients of the pediatric surgery and urology facilities of the Lazio Region. The relevant data have been provided by the Public Health Agency and relate to 2010. The best performance FI has been used to calculate the theoretical volume of transferability of the remaining facilities into freestanding surgery centers. Patients under six months of age and DRGs common to other disciplines have been excluded. The Chi Square test has been used to compare the FI of the health-care facilities and the FI of the places of origin of the patients.

**Results:**

The DRG provided in 2010 amounted to a total of 5768 belonging to 121 types of procedures. The application of the criteria of inclusion have led to the selection of seven final DRG categories of minor surgery amounting to 3522 cases. Out of this total number, there were 2828 outpatients and 694 inpatients. The recourse of the best performance determines a potential transfer of 497 cases. The total outpatient volume is 57%. The Chi Square test has pointed to a statistically significant difference of the facilities and to a non-significant difference of inferiority of the regional places of origin with respect to the city of Rome.

**Conclusions:**

The activity volumes would seem to support the setting up of a Freestanding Regional Center of Pediatric Day Surgery. This Center represents the healthcare facility that is most likely to allow a de-hospitalization process. Subsequent studies will be required to confirm the validity of this pilot study.

## Introduction

The selection of both the type of admission, whether regular or in a day surgery unit, and the continuous-cycle or exclusively daytime facility represents a fundamental aspect in the organization of minor surgery for pediatric patients.

The regular admission should be restricted to patients whose age and clinical conditions contraindicate the recourse to day surgery [1]. The healthcare arrangements are extremely different.

The Lazio Region has identified three basic types of healthcare facilities based on a State-Regions agreement [2,3]. Single or multi-specialist *Integrated Day Surgery Unit*, located within a continuous cycle hospital facility devoted to day care. This type of facility may be likened to the model of the Hospital-based facility of the Anglo-Saxon countries [4]. *Day Surgery Center*, involving a facility exclusively devoted to day-care admissions. *Dedicated nursing beds *within a regular hospital unit when the activity is sporadic. Similar settings are also present in other European countries [5].

Both in Italy and in Europe, the "Day Surgery Centers" - particularly in the pediatric sector - have failed to become as widespread as in the United States of America [US], where, defined Ambulatory Surgery Centers (ASC) or Pediatric Outpatient Pediatric Surgery (POPS), they have increased to a considerable extent. Being these facilities autonomous with respect to hospitals, they are also defined as freestanding surgery centers [6,7].

The purpose of this study is to establish, having recourse to an activity indicator, the regional healthcare volumes provided through day surgery centers and the healthcare volumes provided though regular hospitalization that could be transferred to day surgery centers based on the healthcare facility best performance criterion. The indicator that has been devised (freestanding indicator, FI) measures the outpatient/total patient ratio of the most frequent minor surgery DRGs (Diagnosis Related Groups) handled in 2010 by the pediatric surgery facilities in the Lazio Region.

We believe that the resulting data are likely to prove useful to decision makers for planning subsequent economic and marketing studies, verifying the hypothesis that the setting up of a freestanding inter-hospital pediatric day surgery center may allow the de-hospitalization of most pediatric surgical activities.

## Materials and methods

This preliminary observational cohort study has not required the opinion of the ethical committee nor any informed consent being a population study.

The STROBE-Statement checklist [8] has been taken into consideration for its draft. The study has been conducted in the Lazio Region and the data being dealt with relates to the DRGs produced in 2010 by the regional surgery and pediatric urology facilities. The data were provided on line by the Public Health Agency of the Lazio Region (ASP Lazio).

Outpatients and inpatients of the individual DRGs have been evaluated based on the healthcare providers and the patients' places of origin. The healthcare facilities were the Operating Units (UO) of pediatric surgery and urology of the Lazio Region: Pediatric Hospital "Bambin Gesù" (OPBG, with two separate branches), "San Camillo" Hospital. (AOSCF), "A. Gemelli" General Hospital, "Umberto I" General Hospital. These facilities are located within the city of Rome, with the exception of a branch of the "Bambin Gesù" hospital which is located in the northern area of the Rome Province. The organizational models adopted by these facilities have been studied by means of separate surveys. The study has also taken into consideration the DRGs produced by non-specialized pediatric facilities. The places of origin that have been taken into consideration have been Rome and the Rome Province, Latina and the Latina Province, Frosinone and the Frosinone Province, Viterbo and the Viterbo Province, Rieti and the Rieti Province, as well as cities and Provinces outside the Lazio Region.

A selection of the DRGs has been made having recourse to the FI, a DRG-specific indicator that measures the ratio of outpatients to the total number of treated patients. The numerator is represented by the patients discharged on the same day of treatment; the denominator is represented by the sum of all the treated patients of the DRG under consideration. Therefore, the denominator includes regular one-day admissions (one-day surgery) and admissions for 2 or more days. Assessing the level of elimination of regular hospital admissions and, therefore, of overnight stays, this index points to the percentage of services that may be theoretically provided by freestanding centers.

The criteria for inclusion into the study (with a ranking process) were all the DRGs with an FI exceeding the 3^rd ^quartile present in at least a providing facility, with volumes of outpatients exceeding 20% and a volume of cases exceeding the entire case study by 0.5%. The highest value of the indicator identifies the best performing facility. The criteria for exclusion were all the patients under six months of age and all the DRGs in common with other disciplines.

The DRG-specific FI of the best performing providing facility has allowed calculating the theoretical volume of transferability in freestanding mode of the remaining facilities. The FI was also calculated based on the place of origin of the patients.

The statistical analysis had recourse to the Chi Square test for comparing the FI of the providing facilities and of the various places of origin. The study sample size has been the population being considered, that is 5768 cases. 361 cases would have been enough to calculate the size of a hypothetical random sample for a 95% confidence interval. The power calculated for the test was 80% with a level of significance of 5%. The statistical analysis was carried out with OpenEpi open-source statistic software (Copyright _ 2003, 2008 Andrew G. Dean and Kevin Mt. Sullivan, Atlanta, GA, USA).

With reference to inpatients, the prevalence of a one-night hospital admission (one-day surgery) was evaluated with respect to the admissions for two or more nights. An unspecified number of patients experienced during their hospitalization a change in their planned admission from outpatient to inpatient and vice versa.

## Results

**Figure 1:**
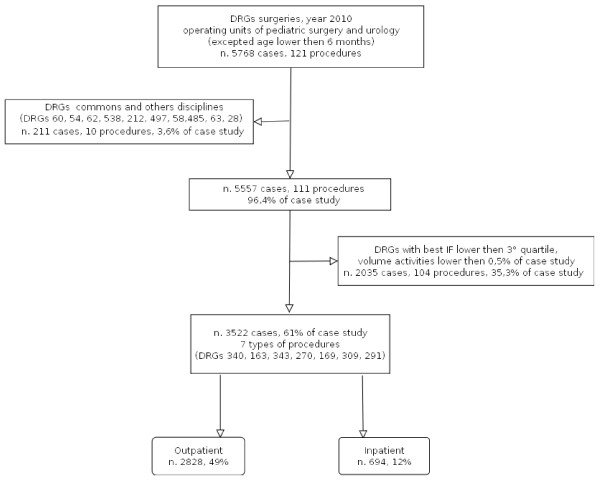
**Flowchart DRGs produced in 2010 by the regional surgery and pediatric urology facilities**.

The DRGs provided in 2010 by the pediatric surgery and urology facilities, with the exclusion of patients under six months of age, amounted to a total of 5768, belonging to 121 types of procedures (Figure 1, flowchart).

The exclusion of DRGs shared with disciplines other than that taken into consideration or with a better performance FI under the 3^rd ^quartile or with volumes of outpatients under 20% or DRGs with volumes under 0.5% of the total case study, has led to the selection of seven final DRG categories of minor surgery amounting to a total of 3522 cases. Out of these, 2828 - equal to 49% of the initial case study - were outpatients, while 694 were inpatients. The weighted average of the FI by DRG typology, corresponding to outpatients, is 81% (Table 1)

**Table 1:** Index of selected Freestanding Drg and volumes of activity.

DRG	description	Outpatient	Inpatient	Freestanding index (weighted avg)	Outpatient/opening drg %	Outpatient/closing drg %
340	Testicular surgery	1337	345	79.5	23.2	37.9
163	Hernia surgery	647	140	82.2	11.2	18.4
343	Circumcision	633	94	87.1	11	18
270	Other skin, etc. surgery	114	43	72.6	2	3..3
169	Mouth, etc. surgery	50	13	79.4	0.9	1.4
309	Minor bladder surgery	32	22	59.2	0.5	0.90
291	Thyroglossal duct surgery	15	37	28.8	0.3	0.4
		2828*	694*	81	49	80.3

Data relating to all facilities.

* sum of all facilities

The application of the best performance criterion has determined a potential transfer of 497 additional cases belonging to the seven selected DRGs, equal to 14.1%. Adding the real volumes to the transferable volumes, it turned out that the total DRGs were 3325. The latter represent 57.5% of the outpatients with respect to the original DRGs, and 95% of the selected final DRGs (Table 2).

**Table 2:** Best performance.

DRG	Description	Outpatient: best performance (%)	Outpatient: transferable **	Outpatient total	Outpatient/operning drg	Outpatient/closing drg
340	Testicular surgery	94.3	249	1586	27.5	45
163	Hernia surgery	91.9	76	723	12.5	20.5
343	Circumcision	97.7	78	711	12.3	20.9
270	Other skin, etc. surgery	93.7	34	148	2.6	4.2
169	Mouth, etc. surgery	93.3	9	59	1	1.7
309	Minor bladder surgery	91.4	17	49	0.8	1.4
291	Thyroglossal duct surgery	91.6	34	49	0.8	1.4
		94.4 weighted	497	3325	57.5	95.1

Volumes transferable to day surgery.

The organizational setup of the pediatric surgery and urology facilities are extremely heterogeneous. Out of the 2828 outpatients, 2825 have been treated in two facilities with an FI of 86.6 and 94.3, respectively. The comparison with the Chi Square statistics of the data relative to these two facilities is highly significant (p < 0.001) from a statistical viewpoint. The ratio of the seven selected DRGs to the total services provided by the healthcare facilities ranges between 40 and 72% (Additional file 1).

The FI calculated based on the places of origin of the patients is quite homogeneous. The Chi Square comparison of outpatients and inpatients between Rome and the others cities and Provinces in the Lazio Region, with the exclusion of Latina and the Latina Province, has not provided statistically significant differences of superiority (p > 0.05). The comparison between Latina and the Latina Province and Rome is statistically significant and the FI is higher for Latina and the Latina Province. The FI for the areas outside the Region is 70.5; the Chi Square comparison between patients from the Lazio Region and those from outside the said Region is highly significant (p < 0.001) from a statistical point of view (Table 3). With reference to the inpatients, the one-day surgery accounts for 33.1% with respect to the DRG being considered.

**Table 3:** Geographical origin of patients.

Origin	Outpatient	Inpatient	IF	Test Chi-square (City of Rome)	p
Rome - City	1302	317	80.4	-	-
Rome - Province	694	139	83.3	2.853	0.091
Latina and its province	194	27	87.7	6.459	0.011
Frosinone and its province	149	42	78	0.481	0.488
Viterbo and its province	92	14	86.8	2.211	0.137
Rieti and its province	59	14	80.8	0.004	0.947
Other provinces	338	141	70.5	20.469	0.000
	2828	694	80.3*		

* weighted average

## Discussion

The application of the exclusion criteria to the initial case study has led to the selection of seven types of procedures that represent 61% of the total DRGs provided by the pediatric surgery and urology facilities of the Lazio Region in 2010. In these DRGs, 80% are outpatients. The best DRG-specific performance determines a theoretical 10% increase. There are no sufficient clinical and organizational data to determine the causes of the high recourse to the one-day surgery, which accounts for nearly 1/3 of all regular admissions. It is quite likely that even a part of these admissions with overnight stay could be transferred to day surgery.

The age group included in the study is represented by patients who are over six months of age. This helps to assess the regional volume of procedures that could be supposedly carried out in a freestanding surgery center using the same minimum age cut-off for treatment of the Ambulatory Surgery Centers [1]. As for patients under six months of age, the literature concurs that newborn babies and premature babies with a gestational age below the 60^th ^week of post-conceptional life [9] should be excluded from day surgery. It is advisable that, depending on the protocols adopted by the individual healthcare facilities, babies whose age is included between two and six months be admitted to a hospital-based day surgery unit or dedicated nursing beds [1]. With reference to the age group referred to above, a total of 588 patients have been treated in 2010 by the healthcare facilities taken into consideration. Most of them have been treated having recourse to a regular admission procedure and, in fact, the FI is very low (14.6%).

The organizational models and the FI of the healthcare facilities are independent. Paradoxically, the model of partial de-hospitalization, represented by day surgery units, features a lower FI than the model of the dedicated nursing beds in departments for acute patients. The difference is statistically significant. The analysis of the causes is complex and the phenomenon highlights the inadequacy of the FI as the only indicator of the quality and appropriateness of the day surgery services. It is quite likely that an improved understanding of these causes could be gained by monitoring the indicators laid down in the regulations of the Lazio Region [2]. As for the latter, the one relative to the measures connected with the dedicated nursing beds model could be an aspect to be kept under observation. Other elements that should be considered include the analysis of costs in the two models and the time analysis of the activity data. In any case, it may be assumed that, given the want of resources, in a forthcoming future the model of dedicated nursing beds in mixed (adult and pediatric) continuous-cycle hospitals could be negatively affected by the progressive greater care complexity of adult patients with respect to pediatric patients. This phenomenon could be heightened when the surgical activity takes place in operating units that are not reserved to day surgery. On the other hand, the obstacles to the development of day surgeries could be less significant in exclusively pediatric hospitals owing to the lower ASA Physical Status grading values and the relative time stability of such conditions with respect to grown-ups. Furthermore, with a view to ensuring ongoing quality, effectiveness and efficiency, the only pediatric hospital - the Bambin Gesù Pediatric Hospital (OPBG) - got prevailingly organized with day surgery units for the patients' stay and specific operating units.

The place of origin of the patients, often considered a factor limiting the recourse to a day surgery if located far away from the healthcare facility, has not conditioned the choice of the type of hospital admission within the Region. This may be inferred from the comparison of the FI with the Chi Square test, which has not proved significant based on the place of origin. Indeed, the data relative to Latina and its Province point to the inferiority of Rome. The case is altogether different when dealing with places of origin from outside the Region, the significance of which gives evidence of an inappropriate use of the hospital as the place of an overnight stay.

The study presents additional limitations.

The FI has been constructed by modifying the indicators used for the assessment of the appropriateness of hospital admissions of the APPRO (Appropriateness of hospital admissions) method [10], the transferability of the volumes of day surgery of the MAAP (Model of Analysis of the Procedure Appropriateness) method [11] and the assessment of the balanced scorecard system performance [12,13].

Until now, such methodologies have been prevailingly used for adult patients.

The APPRO, built by the Public Health Agency of the Lazio Region, is based on hospital discharge cards (SDO) and has partly recourse to an isogravity classification system called All Patient Refined Diagnosis Related Groups (APR-DRGs) [14]. The method has been conceived with a view to assessing the behavior of hospital facilities in the provision of care services characterized by a low complexity to patients who are not affected by a clinically severe illness, reasonably assuming that, in these cases, the regular admission to a hospital is as a rule an inappropriate organizational procedure [15]. The APPRO method is used in particular to calculate the admissibility thresholds (to regular admission) for DRGs with a high risk of inappropriateness [16]. In this method, the DRG-specific hospital or regional threshold of "tolerated inappropriateness" is a proportion where the numerator is the number of regular admissions of patients affected by illnesses with a minimum level of severity > 1 day and the denominator is the total number of regular admissions of patients affected by illnesses with a minimum level of severity to the hospital or the day surgery unit. The APPRO method is used in order to enforce systems of rewards or sanctions.

The MAAP method has been adopted by the Puglia Region to build indicators of the care setting transferability, DRGs with a high volume of cases, from regular hospitals admissions to day hospitals and ambulatory surgery centers. The transferability volume indicator is used when the DRG volume exceeds 0.5% of the admissions for the entire case study.

The assessment of the performance of the healthcare systems has been made on behalf of the Ministry of Health by the Management and Health Laboratory of the Sant'Anna School of Advanced Studies in Pisa through a set of 34 indicators calculated on the SDOs in 2007 and 2008 [17]. The regional health systems were benchmarked having recourse to target and quintiles methods that allow moving from measurement to assessment. These indicators include those that analyze the appropriateness of the surgical services, namely the share of surgical DRGs falling within the essential levels of care (LEAs), provided in day surgery and one-day surgery facilities. The volume of transferability into freestanding centers of the regular admissions of the least performing facilities with respect to the best performance that has been adopted in this study presents a few analogies with the assessment systems referred to above, with the difference that the method of quintiles has been turned into quartiles.

The available data are incomplete and this has not allowed determining the extent of the change of the system of admission from outpatient to inpatient and vice versa. This aspect should be monitored as it could invalidate the day surgery activity. Hence, the assessment of the healthcare results with administrative data has both advantages and disadvantages. The immediate access to data on relatively uniform computer files and the possibility of estimating shares of inappropriateness within the context of selected case records are definite advantages. On the other hand, the disadvantages are due to the uncertainty of the nature of the administrative datum (potentially incomplete, inaccurate, and distorted) and the absence of references to the context where the admission takes place (e.g., social conditions of the patient). It ensues that the inner validity of the appropriateness assessment methods depends on the comprehensiveness and accuracy of the SDO compilation and coding.

Notwithstanding the aforementioned limitations, there are also additional systems that back up the recourse to administrative data. In fact, the FI is similar to the ASC proportion of the pay for performance (P4P) system [18]. Within this context, it is used in a few states of the USA in both adult and pediatric age starting from patients over the sixth month of life. The P4P system produces a rewarding payment mechanism for the best performing facilities [19,20].

The avoidable hospitalization is a subject that is well known to pediatricians. By analogy with the studies of the ambulatory care-sensitive conditions that have led to a radical reduction of the medical admissions to hospitals, a few studies that are likely to promote a partial surgical de-hospitalization should be implemented [21]. In fact, in the surgical field, the comparison between ASC and hospital-based facility is still an open question, besides being quite a controversial point [22-24].

Furthermore, there is also considerable confusion about the meaning of de-hospitalization [25]. For administrative personnel, de-hospitalization merely means the passage of a few types of services from the regular hospital setup to a day surgery unit, regardless of the care setting where the process takes place. On the other hand, one needs considering the type of hospital wards and surgery units and, in particular, their inclusion in continuous cycle or day-only facilities. In our opinion, the real de-hospitalization of day surgery is represented by the provision of that activity in daytime facilities and, therefore, with specific hospital wards and surgery units.

The day surgery cases reported in this pilot study only concern pediatric surgery and urology. They should be considered minimum case studies susceptible of increase, since other specialties are also having recourse to day surgery (e.g., ORL, orthopedics, ophthalmology, digestive endoscopy, etc.). Therefore, a considerable part of the services currently provided in a number of continuous-cycle hospital facilities could be concentrated in a hypothetical inter-hospital health facility. We believe that the positive trend of the outpatient/inpatient ratio with respect to previous years, the significant volumes of real and potential activities highlighted in this pilot study, the abolition of the one-day surgery and the extension of the analysis to other disciplines could further the setting up of a freestanding inter-hospital facility of pediatric day surgery in the Lazio Region.

## Conclusions

The implementation of a healthcare facility does not depend solely on the total activity volumes, even though this is an important aspect. This pilot study could turn into the starting point of subsequent studies focusing on health management (e.g., cost analysis), on the expression of the parents' preferences (e.g., conjoint analysis), on marketing, etc., with a view to evaluating the feasibility of a de-hospitalization of the pediatric day surgery through a freestanding surgery center in a Region where the existing facilities are found within hospitals characterized by a high healthcare complexity and are concentrated within the city of Rome.

## Conflicts of interest

The authors declare that they have no competing interests.

## Authors' contributions

All authors were involved in the conception of the study. GM carried out data extraction. He is the corresponding author of the paper. GM conducted statistical analysis. GM drafted the paper with contributions from the co-authors. All authors read and approved the final manuscript prior to submission.

## Supplementary Material

Additional file 1 **Organisational models of the Child Surgery Operational Units**. Click here for file
