# Supplementary material for: De-hospitalization of the pediatric day surgery by means of a freestanding surgery center: pilot study in the lazio region
Source: Ital J Pediatr. 2012 Feb 1;38:5. doi: 10.1186/1824-7288-38-5 (PMC3327633; doi:10.1186/1824-7288-38-5)
Supplement: Additional file 1 — Organisational models of the Child Surgery Operational Units. [file 1824-7288-38-5-S1.DOC]

Additional file 1. Organisational models of the Child Surgery Operational Units

| Hospital |  | Day Surgery Units | | |  | Dedicated hospital beds | | |  | Accredited beds |  | IF |  | Selected DRG / OU cases % |
| --- | --- | --- | --- | --- | --- | --- | --- | --- | --- | --- | --- | --- | --- | --- |
|  |  | Multi-specialist facilities | Single-specialist facilities | Dedicated surgery unit |  | Dedicated DS unit | Child surgery unit | Dedicated surgery lists |  |  |  |  |  |  |
|  |  |  |  |  |  |  |  |  |  |  |  |  |  |  |
| OPBG Roma |  | ✓ | 🗶 | ✓ |  | 🗶 | ✓ | ✓ |  | 4* |  | 86.6 |  | 68 |
| OPBG Palidoro |  | ✓ | 🗶 | 🗶 |  | 🗶 | ✓ | ✓ |  | 22* |  |  |
| AOSCF |  | 🗶 | 🗶 | 🗶 |  | 🗶 | 🗶 | 🗶 |  | 5** |  | 94.3 |  | 72 |
| Policlinico Gemelli |  | 🗶 | 🗶 | 🗶 |  | ✓ | 🗶 | 🗶 |  | 0 |  | 0 |  | 40 |
| Policlinico UmbertoI° |  | 🗶 | 🗶 | 🗶 |  | 🗶 | ✓ | 🗶 |  | 1 |  | 1 |  | 56 |
|  |  |  |  |  |  |  |  |  |  |  |  |  |  |  |

*Multi-specialist accredited beds both locations OPBG

** Dedicated beds in the inpatient
